# From concept to practice: an analysis of the implementation of the biopsychosocial model in public policies for suicide prevention in Spain

**DOI:** 10.3389/fpubh.2026.1987569

**Published:** 2026-10-29

**Authors:** Jose Antonio Llosa, Isabel Montes-Pintos, Cristina Rea-Rodríguez, Beatriz Oliveros, Sara Menéndez-Espina, Esteban Agulló-Tomás

**Affiliations:** 1 Faculty Padre Ossó, University of Oviedo, Oviedo, Spain; 2 Department of Psychology, University of Oviedo, Oviedo, Spain

**Keywords:** biopsychosocial model, policy implementation, public policy, qualitative policy analysis, social determinants of health, suicide prevention

## Abstract

With the World Health Organization (WHO) estimating 720,000 deaths from suicide each year, the phenomenon of suicide is a priority in public health policy. International policy frameworks highlight the need to adopt biopsychosocial perspectives in order to understand the phenomenon of suicide; however, in practice, it is noted that social components play a minor role in policy design, with a preference for a biopsychological and clinical approach. This paper seeks to answer the question: Does Spanish public policy on suicide prevention operationalize the biopsychosocial model of suicide in its strategic implementation? To answer this question, a documentary analysis of public policies is carried out using the READ method. Current suicide policies in force in Spain are examined at three levels: transnational framework documents, national policies, and regional public policies. In the 15 policies analyzed, a lexicometric analysis is combined with a thematic qualitative policy analysis. The results show that, as one moves down to policies closer to the population, a gap emerges between the discursive use of the biopsychosocial model and its practical application. It is concluded that at the transnational and national policy levels, the biopsychosocial model predominates in the understanding of suicide, while in Spain’s autonomous communities, the psychobiological approach tends to be prioritized over social causes. This has an impact on the types of measures proposed, which have limited intersectional scope, a narrow view of the community-based approach, and prioritize healthcare resources to address the phenomenon of suicide.

## 1 Introduction

The World Health Organization (WHO) (1) estimates that every year, 720,000 people take their own lives worldwide. In Europe, this global phenomenon affects 10.37 per 100,000 inhabitants (2). In Spain, the most recent rate is 8.09 per 100,000 (3). In turn, suicide rates are not distributed evenly across gender, age or geographical area. The WHO (4) notes that suicide rates are higher among men than among women and that this gap widens with age. In Europe, the suicide rate among men stands at 16.87 per 100,000, which is four times higher than that recorded among women. In Spain, the rate for men is 12.11 per 100,000, while for women it is 4.22. It is also observed that contexts with greater economic inequality are at higher risk (4). The WHO’s approach involves understanding the phenomenon of suicide from a multi-causal perspective, based on the biopsychosocial paradigm (5). However, authors such as Rodriguez-Otero et al. (6) argue from a Public Health perspective that, in the design of suicide prevention policies, most attention is paid to related biological and psychological aspects, while the social determinants of health are relegated to the background. This critical perspective is the motivation for this study.

Firstly, suicide is a phenomenon linked to mental health. It is therefore worth providing a brief critical overview of the concept of health, defined by the WHO as: “a state of complete physical, mental and social wellbeing and not merely the absence of disease or infirmity” (7). This broad and general definition already includes mental health as one of its pillars. This approach leaves behind the concept of illness on which previous definitions were based, focusing instead on preventive practice and quality of life, and seeking to transcend the medical model (7). The international organization defines mental health as: “a state of mental wellbeing that enables people to cope with the stresses of life, realize their abilities, learn and work well, and contribute to their community. It has intrinsic and instrumental value and is a basic human right.” (8). The adoption of this perspective implies that the WHO endorses Solar and Irwin’s model of the social determinants of mental health (9).

“At any one time, a diverse set of individual, family, community and structural factors may combine to protect or undermine mental health. Although most people are resilient, people who are exposed to adverse circumstances are at higher risk of developing a mental health condition” (8).

With this perspective, authors such as Petek et al. (10) arrive at very specific conceptualisations of mental health, assuming that it is an integral part of health, with the potential to determine quality of life, and enabling individuals to lead a meaningful life, to interact with their environment and the people within it, and to develop fully as citizens. This approach captures the link between social inclusion and mental health, and with it the political dimension necessary for such an approach. Public policy calls not only for intervention on mental health issues, but also for preventive action addressing the structural causes that determine them (11, 12).

The definition of suicide is not limited solely to completed death, but also relates to preceding processes. Therefore, the concept encompasses completed suicide, attempted suicide and suicidal ideation (13–15).

In the scientific literature, at least two frameworks for understanding suicide coexist. One, which we shall refer to as the psychobiological approach - the predominant one, closely aligned with the clinical-psychological and medical model - and another of a biopsychosocial nature, which takes an integrative approach. The psychobiological approach finds its earliest theoretical foundations in theories such as Kelly’s theory of personal constructs (16), which is intrapsychic in nature and focuses on the internal conflicts that individuals experience at a psychological level. This approach has developed, to a large extent, through clinical psychology and psychiatry, focusing on the correlation between clinical disorders and the phenomenon of suicide. Its development and exploration continue in the literature examining the biological causes of suicide, which gained prominence in the 1990s and has continued to evolve to the present day (17).

However, mental disorder should not be regarded as the exclusive cause of suicide, but rather as a correlate of suicidal behavior within a complex web of interacting factors of various kinds (18). This is where the second line of analysis comes to the fore, originating in Durkheim’s classic treatise (19). This approach views suicide as a complex phenomenon with a heterogeneous aetiology, which must be understood in terms of the individual’s relationship and interaction with factors such as culture, religion, social integration, economic changes or even social norms, insofar as these act as forces that regulate and provide the means of expression and the meanings which subsequently enable the assessment of emotions, attitudes and behaviors (20).

This approaches suicide from a biopsychosocial perspective as a historically, socially and culturally determined phenomenon, which explains why rates and prevalence figures vary over time and across regions (21). These variations have been studied by a number of authors from different perspectives in recent years: on the one hand Yur’yev et al. (22) observed that suicide rates are linked to the economic situation and labor market of countries. Along the same lines, Alvarez-Galvez et al. (23) have shown that in Spain, suicide rates stabilized during the crisis, before subsequently rising again with the economic recovery. Other authors such as Choi (24) have found a link between poverty and trends in suicide rates among older people, a finding that can be extrapolated to other age groups. Similarly, Jung & Park (25) found a relationship between housing and the phenomenon of suicide. Other authors also highlight the relationship between suicide rates, working conditions and social relationships (26–30). Finally, a link has also been identified between isolation and suicide (31). This evidence allows the biopsychosocial approach to suicide to be associated with the model of social determinants of mental health (32). Exploring this perspective in further depth, Fernández-Vilas et al. (33) highlight the need to conduct a study of suicide at three levels of analysis: a micro-level relating to individuals’ immediate circumstances; a meso-level examining the determinants of those immediate circumstances or of the individual; and a macro-level enabling the identification of the structural causes that reproduce the meso-level determinants.

When examining the connection between conceptual and etiological perspectives on suicide and their translation into public policy, it is observed that mental health policies in Europe take as their starting point the framework proposed by the WHO (12, 34). This framework promotes three fundamental strategic aspects: firstly, the approach to mental health must be biopsychosocial and evidence-based, thereby moving away from the dominant biopsychological and clinical approaches found in the scientific literature; secondly, it adopts and promotes a paradigm of social determinants of health (9); thirdly, public policies must be intersectoral in nature. From these three elements, other consequences follow, such as the priority given to a community-based approach, the predominance of preventive approaches at all levels, and the need to provide high-quality resources to address mental health needs. After the COVID-19 pandemic, the WHO updated its framework for action on mental health in Europe, based on the same principles, and incorporated a key strand on loneliness and care policies - a European priority arising from its ageing population. Furthermore, a strategic approach to suicide prevention was set out (5):

“Develop, implement and monitor comprehensive multisectoral plans for the prevention of self-harm and suicide among younger people, including enhanced surveillance and follow-up of individuals who harm themselves or attempt suicide, as well as capacity-building among general health-care and community workers” (p.25).

The WHO’s framework for Europe is being rolled out in a cascade effect across the European Union’s mental health policy (35), and consequently across the various member states and their territories. In the case of Spain, the current mental health plan is the National Health System’s Mental Health Strategy for the period 2022–2026 (36).

In recent years, in light of the significance of suicide as a public health issue, mental health plans specifically targeting suicide have been developed. The principles underpinning these suicide prevention plans are also included in the action plan of the WHO’s strategic approach (5). In Spain, the Suicide Prevention Action Plan (37) has been approved. This plan is based on a comprehensive and cross-cutting approach to the development of all health policies, creating an institutional committee in collaboration with the autonomous communities (ACs) to monitor and develop strategic actions relating to suicide prevention. Spain is administratively divided into 17 autonomous communities. Each has an elected president, its own government, and powers over key areas for the regions, such as healthcare systems. The autonomous communities, some having done so previously and others under the umbrella of the national action plan, have been developing their own specific strategies for suicide prevention. This level is particularly relevant, as in Spain it is the administrations of the autonomous communities that have responsibility for healthcare (38). They are, therefore, the administrative body closest to the public and the one with the greatest capacity for action in this sphere.

Despite this, it is evident that many European countries do not have an adequate response to mental health issues. There is a trend whereby countries with a low risk to the mental health of their populations have more effective response mechanisms than those with a high risk of mental health problems. In Spain, a moderate risk is combined with a poor response, in both social and healthcare settings (Figure 1) (39).

**Figure 1:**
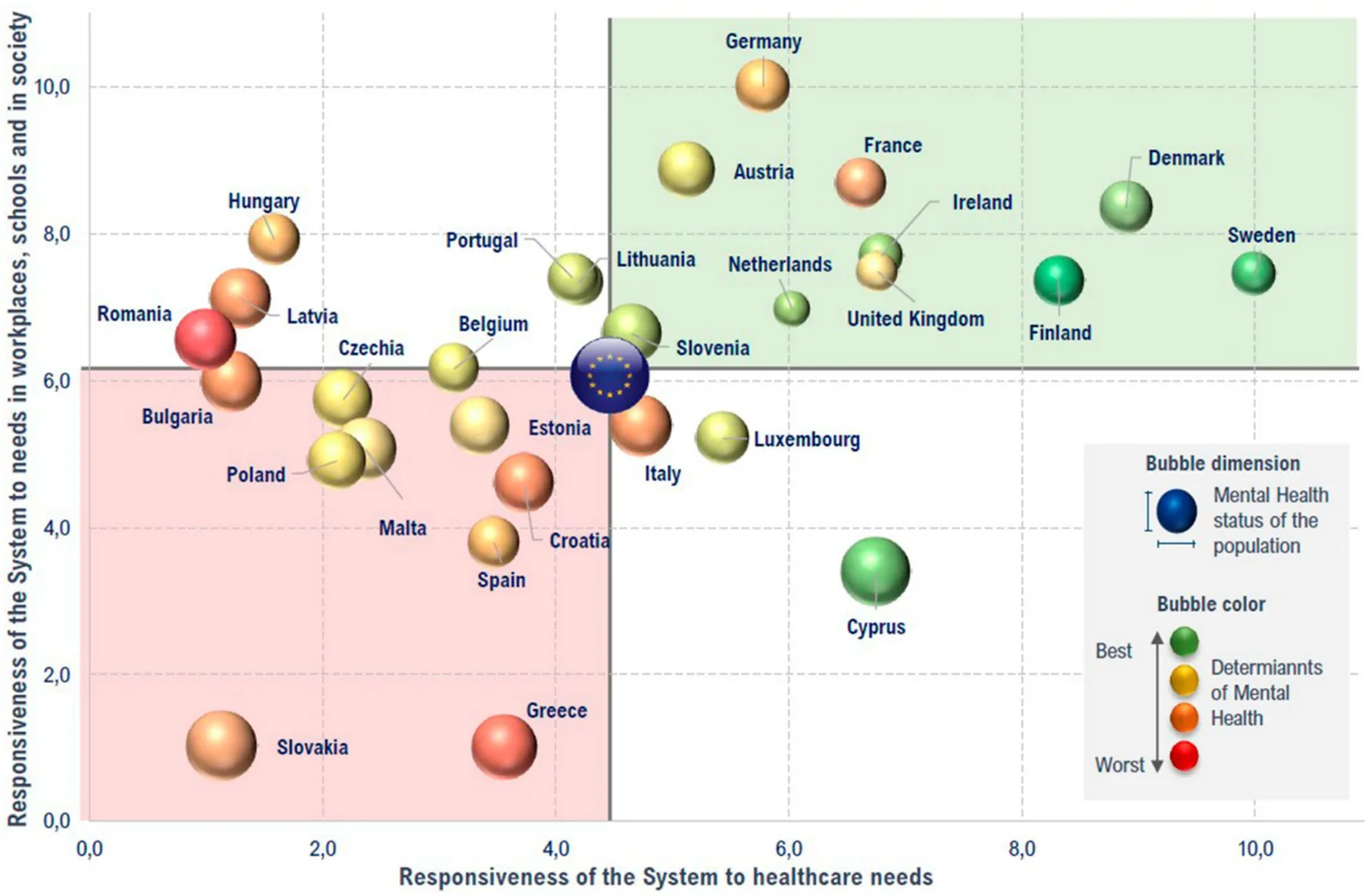
Relationship between the level of risk to mental health and the capacity to respond to mental health needs in European Union countries. Source: Arango et al. (39).

Several reasons have been identified for why the response to mental health issues is not optimal: (1) the first of these is a lack of investment in these healthcare policies (39). Secondly (2), there is a significant difficulty in achieving the intersectoral or multisectoral approach proposed by transnational policies (10). On occasion, this intersectoral approach translates into national-level policies, as in the case of the Suicide Prevention Action Plan in Spain (37), but it is rarely put into practice at a more local level (in autonomous communities or local councils). This is due, among other reasons, to (3) a poor etiological definition of mental health, and by extension of suicide (10). Finally, and linked to this lack of a specific etiological definition, (4) a literature review of European public policies has revealed that countries are more inclined to develop community-based initiatives than legislative measures (40). It is worth setting out a conceptual definition of the community-based approach, namely one aimed at empowering citizens and fostering their autonomy, with the aim of building strong communities that enable spaces of shared responsibility for wellbeing. These are approaches with the capacity to bring about transformative change in the immediate environment, but which are directed towards co-created regulatory and legislative frameworks that define and redefine social structures (41, 42).

While there is abundant literature on the importance of biopsychosocial factors in understanding suicide, and transnational and national policy frameworks tend to adopt this perspective, clear differences remain regarding how effectively these frameworks are translated into the design of proximity policies. Through an examination of public policies on suicide in Spain, this study aims to provide an analysis of this issue, assessing whether the biopsychosocial model is effectively integrated into suicide policy, or whether a narrower psychobiological and clinical-psychological model prevails in its approach and conception. Accordingly, this study is based on the following research question: Does Spanish public policy on suicide prevention operationalize the biopsychosocial model of suicide in its strategic implementation? The study is based on the hypothesis that a psychobiological and clinical perspective predominates in the understanding of suicide, which is reflected in public policy on suicide prevention (6). This poses a risk to the policy’s effectiveness and rationality, as highlighted in the works of Petek et al. (10) and Arango et al. (39).

This question is materialized through two objectives: (1) To determine the theoretical and ideological representation of the concept of suicide as set out in Spain’s public policy on suicide prevention. (2) To analyze the implementation of the biopsychosocial approach in the preventive and intervention measures of Spain’s public policy on suicide prevention.

## 2 Materials and methods

### 2.1 Approach, scope and design

To answer this research question, a qualitative approach is used, which takes the form of an analysis of suicide prevention and intervention policies specific to the Spanish context: this includes suicide prevention and intervention policies at European, national (Spain) and autonomous community levels. Two perspectives coexist in the development of this methodology, which in this research will be combined and reconciled. A small q perspective, with a positivist foundation and supported by techniques such as lexicometric analysis, alongside a big Q orientation, more typical of constructivist approaches, for an analytical interpretation of textual data characteristic of qualitative policy analysis (43). According to Brady et al. (44) This work is developed methodologically along a third path that he calls dualist, representing the combination of both perspectives: small q and big Q. This combination of several methodological techniques seeks to reduce possible bias in each one of the approaches separately (45).

The specific procedure for conducting this policy literature review follows the READ framework (45). This summarizes the research process in four steps: R (ready your materials); E (extract data); A (analyze data); and D (distil your findings).

### 2.2 Sample

The first phase of the READ method involves constructing the sample of documents (ready your materials): following Martínez de Sánchez (46), a non-probabilistic, purposive sampling approach was used to construct the document sample. The attributes of the sample are based on Burch’s conceptual framework for public policy (47), which articulates the levels of public policy affecting a territory within a logical-rational structure linking the political, strategic, tactical and operational levels (47). For this study, strategic documents have been selected as the appropriate level of analysis; these generally take the form of plans or strategies approved at the transnational level for implementation within the European Union (EU/WHO), at the national level (Spain) or within Spain’s autonomous communities. The attributes or criteria of the sample selected across the three levels are set out in Table 1.

**Table 1:** Attributes or criteria of the selected sample of documents.

Transnational level (European Union/WHO)
Strategic documents drawn up by the European Union, the implementation of which includes Spain. These comprise strategies, strategic frameworks and communications from the European Commission. WHO strategic guidance documents applicable to the European region. Thematically linked to planning in the field of mental health in general. Documents which, while addressing health, do not include measures specifically aimed at mental health will not be considered. Dating from within the last 15 years. Their application status must be current. Current does not mean that their application period has ended, but that there is no subsequent strategic framework replacing them. Consistent with the objective of the study. Available in Spanish or English.
National level (Spain)
Strategic documents developed by the Spanish Government. This includes strategies and strategic frameworks. Thematically linked to planning in the field of mental health in general. Documents which, while addressing health, do not include measures specifically aimed at mental health will not be considered. Dating from within the last 15 years. Their application status must be current. Current does not mean that their application period has ended, but that there is no subsequent strategic framework replacing them. Consistent with the objective of the study. Available in Spanish or English.
At the level of Spain’s autonomous communities
Strategic documents that have been developed by any of the Spanish autonomous communities. This includes strategies and strategic frameworks. Thematically linked specifically to the field of planning for the prevention of or intervention in suicide. Dating from within the last 15 years. Their application status must be current. Current does not mean that their application period has ended, but that there is no subsequent strategic framework replacing them. Consistent with the objective of the study. Available in Spanish or English.

To identify documents for inclusion in the analysis, a search strategy was followed using both generalist and institutional search engines: Government of Spain and EU. To identify national or European documentation, the following search string was used: (“Estrategia” OR “Plan” OR “Strategic”) AND (“Salud Mental” OR “Mental Health” OR “Suicidio” OR “Suicid*”). When seeking to identify documents relating to the Spanish autonomous communities, the following search string was repeated for all Spanish autonomous communities. Andalusia is used as an example: (“Andalucia”) AND (“Estrategia” OR “Plan” OR “Strategic”) AND (“Salud Mental” OR “Mental Health” OR “Suicidio” OR “Suicid*”). Similarly, a targeted search was carried out on the corporate websites of the regional governments where no strategic documents had been identified using the search engines mentioned above.

Once the searches had been carried out and the attributes applied, the study yielded a sample comprising 15 documents. Of these, 3 are of European scope (transnational level), 2 are national, and 10 relate to the Spanish autonomous communities (regional level). In terms of language, 1 document is in English and 14 in Spanish. The publication dates of these documents range from 2017 at the earliest to 2025 at the latest (Table 2).

**Table 2:** Sample of documents analyzed.

Group of documents	Document Type	Territory	Document title	Document date
Transnational	Mental health	European	European Commission on a comprehensive approach to mental health	2023
Transnational	Mental health	European	WHO European framework for action on mental health	2022
Transnational	Mental health	European	WHO An implementation guide for suicide prevention in countries	2021
National	Suicide	National	Action Plan for Suicide Prevention	2025–2027
National	Mental health	National	Mental Health Strategy of the National Health System	2022–2026
Regional	Suicide	Andalusia	Suicidal Behavior Prevention Program in Andalusia	2023–2026
Regional	Suicide	Aragon	Suicide Prevention Strategy in Aragon	2020
Regional	Suicide	Balearic Islands	Plan for the Prevention, Response, and Management of Suicidal Behavior	2022
Regional	Suicide	Castile-La Mancha	Suicide Prevention Strategies in Castile-La Mancha	2018
Regional	Suicide	Castile and León	Suicidal Behavior Prevention Strategy in Castile and León	2021–2025
Regional	Suicide	Basque Country	Suicide Prevention Strategy in the Basque Country	2019
Regional	Suicide	Extremadura	Extremadura’s Second Action Plan on Suicidal Behavior	2021
Regional	Suicide	Galicia	Suicide Prevention Plan in Galicia	2017
Regional	Suicide	Madrid	Suicide Prevention Plan for the Community of Madrid	2022–2026
Regional	Suicide	La Rioja	Suicide Prevention Plan in La Rioja	2018

There are no policy documents for Asturias, the Canary Islands, Cantabria, Navarre or Murcia, as they do not currently have a strategic-level document specifically aimed at the prevention and/or intervention regarding suicide. It has not been possible to access the document for the Valencian Community. The Catalan strategy is not available in Spanish or English; consequently, it is not feasible to include it in the study in accordance with the aforementioned criteria.

### 2.3 Data analysis

Following the READ method, data analysis comprises two phases in this study: data extraction and information analysis.

With regard to data extraction, two techniques were used: one aimed at lexicometric analysis (small q) and the other at qualitative policy analysis (big Q). For the lexicometric analysis, text mining was applied to the extracted documents to carry out automated data extraction using MAXQDA 24.10.0 (48). One of the challenges of this documentary analysis is that the texts are in two languages: Spanish and English. While both languages were included in other phases of content analysis, for the lexicometric analysis it was decided to include only the texts in Spanish.

With regard to data extraction for thematic qualitative policy analysis, an inductive coding system was followed. Drawing inspiration from the grounded theory procedure (49), the first phase of the coding process involved a general reading of the text. This led to the emergence of the first draft of the codebook for information processing through open coding. The codebook was refined throughout the data analysis process through the incorporation of in-vivo codes. Finally, the corpus of codes included in the analysis was organized into categories and subcategories through an axial coding procedure (50). This entire analysis was also carried out using MAXQDA 24.10.0 software (48).

As a result of this inductive working process, a series of thematic axes were established to organize and structure the codes for thematic qualitative policy analysis (51):

- Concept of mental health: Aspects relating to the technical, ideological and political perspectives on mental health within the public policy framework concerning suicide.
- Conceptual framework of suicide in public health policies. Etiology, characterization, understanding and/or perspective on the phenomenon of suicide, as an element of specific importance in mental health prevention and intervention within the framework of public policies.
- Priorities in public policies regarding mental health: Identifying priorities in mental health prevention and intervention, paying particular attention to the development, strengthening and funding of resources, services or initiatives.
- Suicide intervention strategies. Approaches to developing intervention proposals addressing the phenomenon of suicide within related public policies.
- Suicide prevention strategies. Approaches to the development of preventive proposals concerning the phenomenon of suicide that are implemented through related public policies.

With the data extracted and coded, the next methodological phase of the READ method (45) is the analysis strategy. With regard to the lexicometric study, three different types of analysis were established with the aim of examining the texts: analysis of term repetition frequencies; term co-occurrence; and relationships between tokens (52, 53). This lexicometric analysis revealed trends and dynamics that guided and enhanced the depth of the thematic qualitative policy analysis.

With regard to qualitative policy analysis, a thematic analysis was carried out based on the themes outlined above, adopting a critical perspective that enabled the extraction of meanings, signifiers and frameworks from the conceptual and contextual framework under analysis. This facilitated the construction of the social phenomenon under investigation (49).

## 3 Results

### 3.1 Theoretical and ideological representations of suicide in public policy

This analysis addresses objective number 1 of the study: to determine the theoretical and ideological representation of the concept of suicide as set out in public policy on suicide prevention in Spain.

By comparing the three policy levels examined in the documentary analysis - from a transnational perspective (WHO and the European Union), to a national perspective (the Spanish Government), and to perspectives closer to the local level (Spanish autonomous communities) - it can be seen that the approach to mental health and suicide is explicitly biopsychosocial (Figure 2). Indeed, this is clearly set out in the European framework documents:

**Figure 2:**
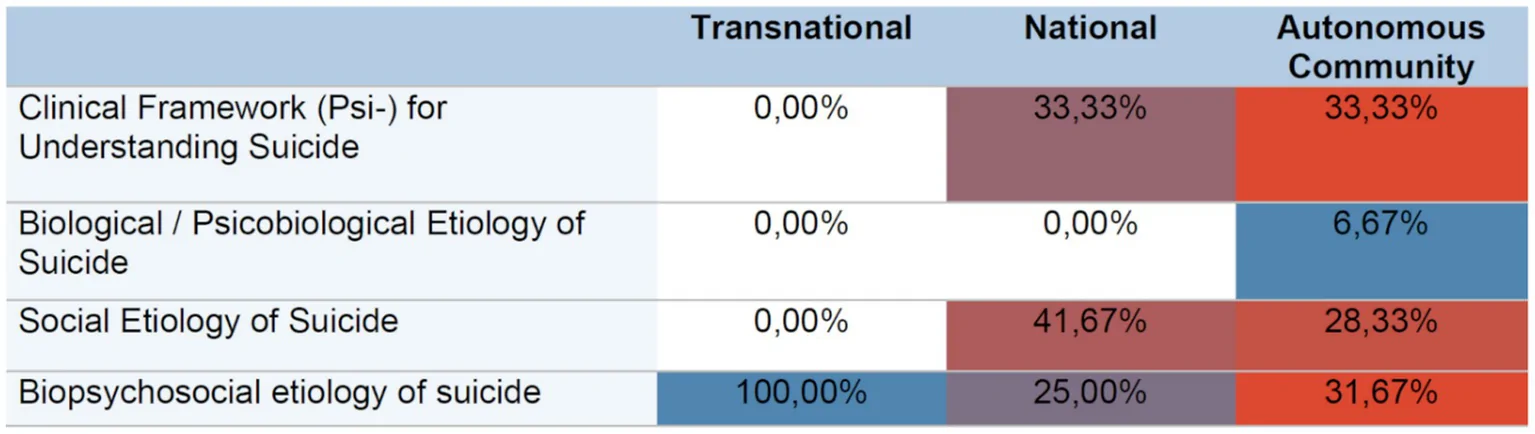
Occurrence of codes in document groups.

“Mental health and well-being are put at risk by a wide range of factors spanning individual, social and environmental levels, including poverty and deprivation; debt and unemployment; and violence and conflict” (5) (Transnational level).

However, if we examine the frequency of occurrence of codes at the different policy levels analyzed in the documents, it becomes apparent that at the national and regional levels, the biopsychosocial etiology is not even the most frequent code (Figure 2). In fact, there is a decreasing trend in the biopsychosocial approach as policy becomes more locally focused: the occurrence of codes relating to biopsychosocial etiology within the transnational framework is 100% in the understanding of suicide; at the national level, it stands at 25%, alongside 41.67% relating to the social etiology of suicide. In the ‘etiology’ category within state policy and strategic documents, there is a strong social component, but a clinical orientation to the understanding of suicide also emerges. At the regional policy level, with the highest proximity, biopsychosocial etiology accounts for 31.67% of code occurrences, social etiology for 28.33% and, for the first time, biological etiology of suicide appears with a 6.67% occurrence rate. There is no trace of biological etiology in the understanding of the phenomenon of suicide within the transnational or national political frameworks.

The key to the biopsychosocial perspective lies in the fact that the three elements (biological, social and psychological) interact, resulting in a unified concept that represents more than the sum of its parts. “It is important to consider the interaction of these factors and to understand them from the perspective of integrating vulnerability factors (characterized as being of a psychobiological nature) with risk factors (defined as being of a psychosocial nature)” (36) (National policy level).

In fact, in the transnational policy framework, the etiological approach connects with the need for transformation and the mutual influence between mental health and the economic and productive sphere: “Mental health is a precondition for a productive economy and inclusive society and goes beyond individual or family matters” (35) (Transnational policy level). However, qualitative policy analysis reveals that in Spain, mental health is increasingly viewed in terms of illness or disability: “With the entry into force in our legal system, on 3 May 2008, of the UN Convention on the Rights of Persons with Disabilities, mental health issues are addressed as a human rights issue and from a perspective dominated by the biopsychosocial model” (36) (National policy level). When mental health is viewed from these perspectives, it tends to be reduced to psychobiological etiological postulates; and if specifically framed in terms of disability, to conceptions of mental health characterized by chronicity.

Spain’s National Health System Mental Health Strategy 2022–2026 (national policy level) sets out a specific strategic line of action for suicide (Strategic Line 3). The introductory section of this line of action proposes a comprehensive approach to the phenomenon:

“Not all suicidal behavior is an expression of a mental disorder. However, it is known that people suffering from mental disorders die by suicide at a much higher rate than the general population. The proportion of completed suicides associated with mental disorders is around 80-90% (there are mental health problems that contribute to the development of suicidal tendencies)”. (36) (National policy level).

This approach neither addresses nor questions the fact that the mental disorders mentioned may be either a precursor or a cause. If they are understood as a precursor, one might ask what structural social factors are determining them. Secondly, the conceptualization overlooks a clear definition of the boundaries of suicide beyond the act of taking one’s own life. There is no specific focus on other components of the phenomenon of suicide, such as suicidal ideation, which has great potential for prevention. This is despite the fact that the strategic and tactical focus of this line of work is primarily oriented towards preventive approaches.

The text goes on to present an etiological approach to the phenomenon, which is of a psychobiological nature and characteristic of the clinical field of psychological sciences: “the issue that appears to be common in suicide is the existence of great emotional suffering” (36). The phrase “appears to be” should be noted as it is non-specific. The same national-level text also assumes that the etiology of suicide is not known with certainty and that preventive approaches (primarily social) appear to be the most appropriate strategy.

If we analyze the direction taken by policies in Spain’s autonomous communities, we observe that biological and clinical approaches are more prevalent in explaining the etiology of the phenomenon of suicide. Biological etiological classifications account for 6.67%, displacing ideas based on etiological postulates related to social causes and determinants of health.

In particular, the policy discourse in autonomous communities reveals a tendency to address suicide based more on technical than conceptual approaches: it avoids the need to define a detailed etiology for the phenomenon of suicide, limiting it to an explanation based on collections of vulnerability factors that may have been detected around these phenomena. In light of this stance, three appreciations can be made:

- The complexity and ongoing debate regarding the causes of suicide within the scientific community lead to a conceptual definition of the phenomenon that is rather vague in public policy. This reveals a tendency to adopt hegemonic etiological assumptions, which in the case of suicide and mental health in general, tend to neglect social causes.
- Secondly, and in connection with the previous point, both the etiological framework and the intervention and prevention strategies of the autonomous communities are closer to psychobiological than biopsychosocial postulates. This aligns with the hegemonic scientific literature emerging from the psychological sciences, rather than with the perspective and approach of European policy, which is based on a decidedly biopsychosocial approach both conceptually and in its application.
- Thirdly, the intervention and prevention proposals are more clinical and care-oriented in nature than transformative. As the understanding of mental health moves in this biopsychological direction, the implications for intervention become evident, reflecting a dualism between the biomedical approach that cures or alleviates; and the social approach, a strategy for support or prevention, but without the potential to resolve mental health problems. This is reflected at the national policy level: “a person can regain their life (social recovery) without necessarily having recovered from their health problem (clinical recovery)” (36) (National policy level).

### 3.2 Implementation of the biopsychosocial approach in the preventive and intervention measures of public policy on suicide prevention in Spain

These findings are linked to the second objective of this study: to analyze the implementation of the biopsychosocial approach in the preventive and intervention measures of public policy on suicide prevention in Spain. Specifically, the aim is to ascertain whether the social component, through the social determinants of mental health, is present and addressed in the measures implemented in Spain.

Firstly, the Spanish Government’s Suicide Prevention Action Plan identifies initial limitations (37): (1) a lack of data sources; (2) a weak culture of evaluating the guidelines and strategies applied; and (3) limited capacity for intersectoral coordination. However, the WHO’s guidelines for suicide prevention in countries directly address these critical issues through an approach known as LIFE (54): (1) restricting access to means of suicide; (2) promoting responsible reporting in the media; (3) prioritizing prevention among young people; and (4) early identification of all those affected. To this end, they identify as guiding principles the need for further analysis, the importance of awareness-raising and training, surveillance and monitoring, adequate funding for policies and, most crucially, multi-sectoral collaboration.

The transnational framework for suicide prevention and intervention, transposed into Spanish national policy, relies on the autonomous communities for its implementation. In Spanish health policy, it is the autonomous communities, the administrative structures closest to the population, that have the authority to deploy healthcare services. Based on these premises, three elements of the policies of Spain’s autonomous communities are analyzed in relation to this objective: intersectoral action, preventive measures, community-based approaches and transformative ambition, and intervention measures.

The analysis shows, first of all, that all public policies linked to suicide in Spain, both at national and regional level, are proposed by health policy bodies, be they the Ministry or the regional health departments. The only autonomous community with a specific body for suicide monitoring is the Balearic Islands, which has a Suicide Observatory. As such, it is also the community with the clearest commitment to an intersectoral approach among those studied. This is evident in proposals such as: “the formation of specific working groups for suicide prevention, coordinated and led by the Suicide Observatory, involving professionals from different areas of care” (55) (Autonomous Community Policy Level). In this context, there is discussion of leadership and coordination capacity. In the remaining regional strategies and plans, 100% do indeed address intersectoral action, but more as a principle or ideal than as a planned and concrete strategy to be implemented. In this regard, measures are presented that primarily relate to standardized procedures for cases where a risk of suicide is detected, or the training of relevant professionals; however, there is no clear indication of which body or bodies are responsible for and authorized to lead this process of intersectoral or multisectoral coordination. This allows the intersectoral approach to tackling suicide to be traced from transnational to national policy as a clear priority, but at the regional level it is presented more as a desirable political goal than as a measure with a realistic prospect of being implemented.

In order to understand the political approach to suicide intervention and prevention, it is necessary to link this to the conceptual and etiological frameworks previously analyzed. An analysis involving the mapping of relationships between codes identified in the policy documents of Spain’s autonomous communities shows that etiological causes are linked to clinical prevention and intervention strategies, while biopsychosocial etiological approaches or those based on social determinants of health are linked to social and community-based prevention and intervention strategies (Figure 3).

**Figure 3:**
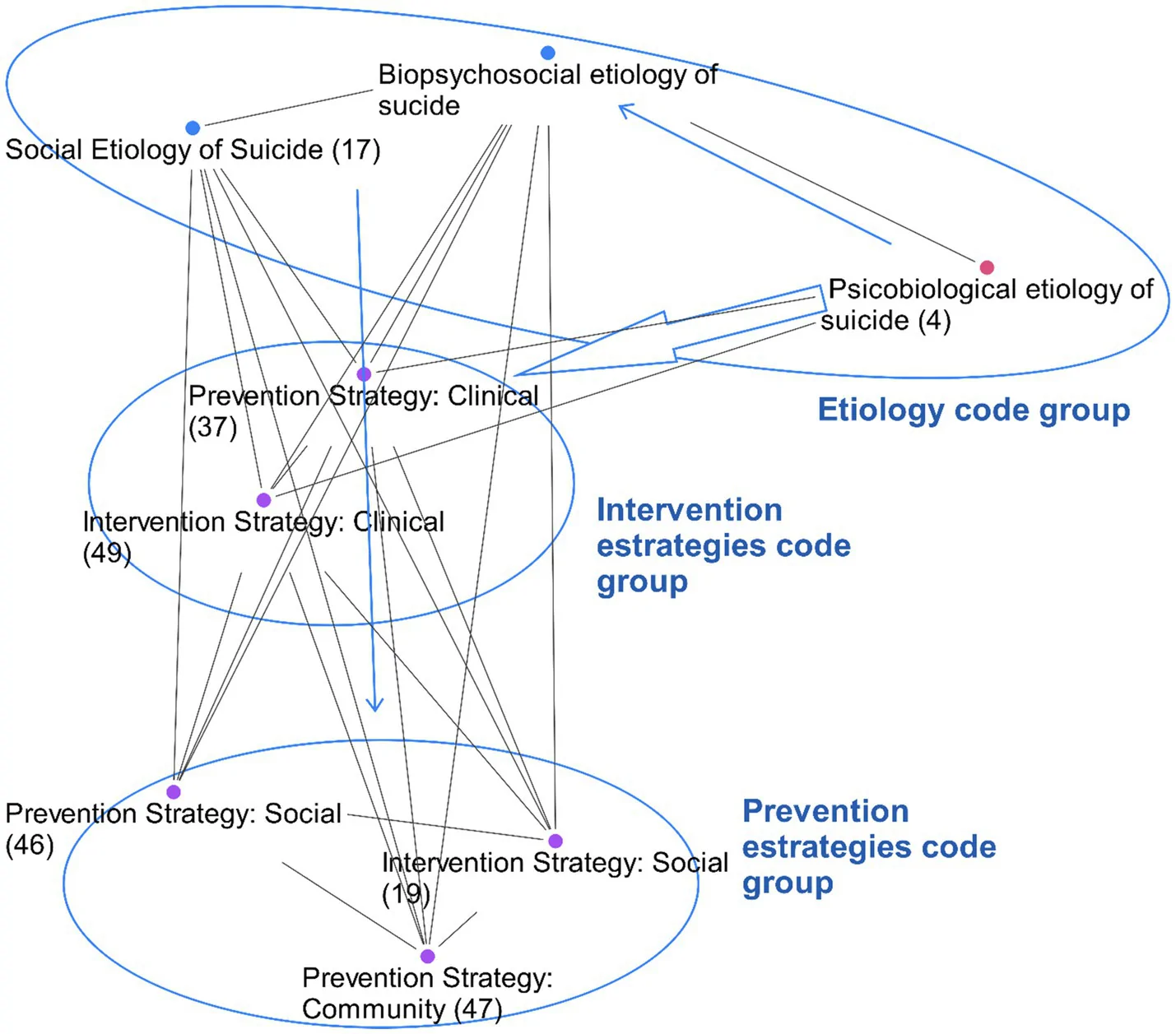
Link between etiology and preventive and intervention proposals in regional suicide policy.

This idea is reaffirmed and explained in the Suicide Prevention Action Plan (37), which offers a rather non-specific approach to the causes and factors involved in the phenomenon of suicide. It presents it as a generally emotional phenomenon, which affects certain social groups in a specific way:

“In other words, these (explanatory) factors have proved to be of limited accuracy in predicting who might develop suicidal thoughts or behavior. No broad category of factors has demonstrated robust predictive power, and studies have rarely investigated how various risk factors interact with one another, which limits the scope of the research” (37) (National policy level).

Given the limited etiological specificity of the policy approach, the thematic analysis reveals a tendency towards a level of care-based intervention focused on contexts of immediate risk to the individual - for example, settings such as schools, or groups such as older people - rather than at a meso or macro level addressing the structural social causes of the phenomenon of suicide or the factors that perpetuate the risk.

In fact, the lexicometric analysis shows that, across the set of public policies analyzed at all levels, the term ‘suicide’ has the strongest association with the token ‘prevention’. In this context, it appears 1,260 times. This is double the number of the next most common related term: ‘risk’ (Figure 4). This exploratory approach highlights a fundamentally preventive focus in the intent of health policies on suicide.

**Figure 4:**
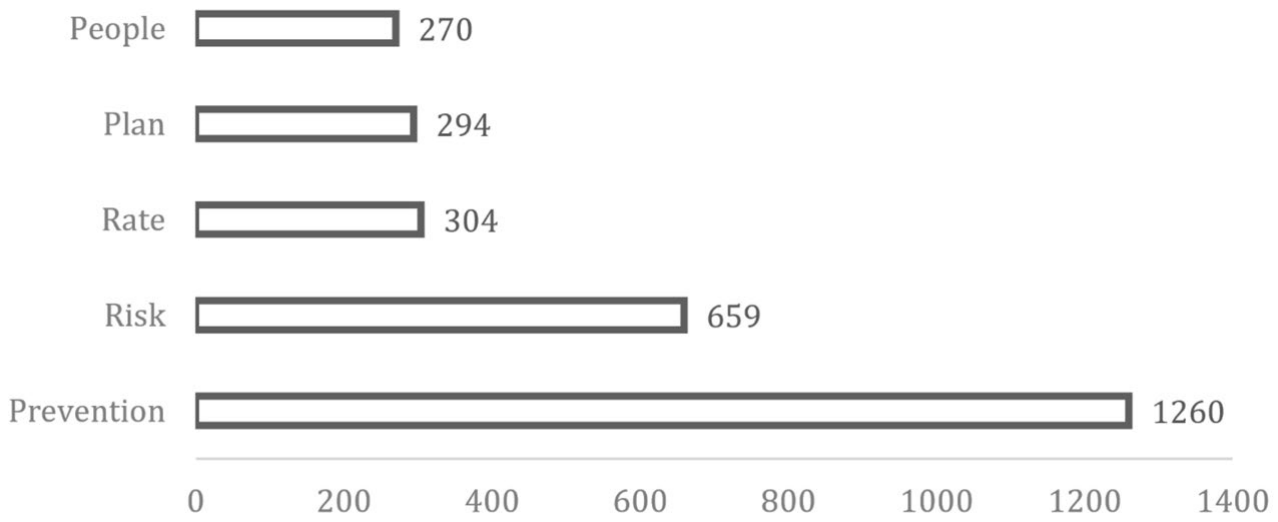
Analysis of frequent contexts for the term ‘suicide’ across all project documents.

Similarly, among the proposed preventive measures, it is common to call for a community-based approach in regional policy documents. Upon closer examination of the characteristics of this approach, it is seen to be limited to the need to involve actors and spaces within the communities themselves (primarily schools, specialized social entities, pharmacies and social services), but more in a role focused on raising awareness or providing information than on seeking to transform social models or conditions. Suicide prevention strategies linked to reducing inequality and poverty, or to tackling racism, homophobia or xenophobia, or to transforming the labor market, feature only marginally in the envisaged community-based approaches.

The focus narrows further when examining measures aimed at intervening when faced with the phenomenon of suicide, rather than at prevention. In the event of a suicide attempt, the policy focus is first on medical care, and then on clinical and psychological support:

“To ensure that the discharge report from the A&E department is forwarded to the primary care doctor (with the necessary guarantees of confidentiality and professional autonomy) in order to ensure the continuity of the necessary care, as well as to the Mental Healthcare Centre” (36) (National policy level).

In other words, it appears that public policy on suicide is showing an interest in the relationship between the social context and the phenomenon of suicide because the etiology of the phenomenon is unclear. When the situation regarding suicide is clearer, as is the case with suicide attempts, policy recommendations tend to confine measures to traditional healthcare settings. The underlying assumption in the measures studied is that the clinical approach, based on psychobiological perspectives, cures, while the social approach merely prevents. It follows from this that the social component is not regarded as a structural cause of the phenomenon of suicide.

## 4 Discussion

The research question of this study was: Does Spanish public policy on suicide prevention operationalize the biopsychosocial model of suicide in its strategic implementation? As the main finding in this regard, the analysis indicates that the biopsychosocial perspective is reflected in the approaches of transnational policy, and is also present in the principles of Spain’s national suicide policy; however, as the policy moves into contexts closer to the population, such as Spain’s autonomous communities, the tendency in public policies to address suicide shifts towards approaches that take precedence over the psychobiological understanding of suicide.

This is consistent with the initial hypotheses and the view that social components are neglected in the planning of public policies on suicide (6). It also aligns with the inherent limitations of the biopsychosocial model, which in its policy and practical application, tends to fragment into its three components, subordinating the explanatory power of the social component to psychologistic or biologistic assumptions (56). This thus addresses objective number 1 of the study, which aims to explore the theoretical and ideological representation of the concept of suicide in Spanish public policy.

Conceptually and theoretically, it can be observed that transnational policy, whether directly formulated by the WHO or highly determined by its principles, is linked to the WHO’s models of the social determinants of health (9). When analyzing the policies of the autonomous communities, however, the understanding of the phenomenon of suicide leans more towards biomedical approaches than biopsychosocial ones (56, 57). In many cases, the comprehensive or conceptual understanding of the phenomenon of suicide is absent from the preambles of policy documents on suicide in the autonomous communities, implying a tendency to adopt hegemonic assumptions in the understanding of mental health. These assumptions are historically linked to biomedical perspectives (58–60).

Consequently, transnational and national policy approaches recognize that the causes of suicide are also linked to structural issues, and call for a transformation of the population’s living conditions as a means of preventing and intervening in cases of suicide (5, 36, 37). These principles are consistent with trends in the scientific literature (6, 23, 61). However, when examining suicide prevention policies that are more locally focused, and therefore with greater capacity for deployment, their measures tend to be more welfare-oriented than transformative, substantially blunting their effectiveness. This trend has been observed in health policy at the international level, not only in the Spanish context (10, 40). Thus, in Spain there is a contradiction between a transnational and national policy approach with transformative ambitions which, when it reaches the public, is limited to a set of welfare-oriented measures.

The second objective of this study was to analyze the implementation of the biopsychosocial approach in the preventive and intervention measures of Spain’s public policy on suicide prevention. This is developed by focusing on social determinants, as this conceptually constitutes the social element of the biopsychosocial model. The WHO’s LIFE paradigm on suicide (54) emphasizes the importance of intersectoral frameworks for strategically addressing suicide (5, 35). However, when this fundamental principle is applied at the local or regional policy level (autonomous communities), it is reduced to superficial measures such as training programs offered to professionals from different sectors of the administration, or the formulation of protocols for the coordination of a diffuse structure, governance and viability. In all the regional cases studied, with the exception of the Balearic Islands, suicide prevention policies are promoted by health departments (or their equivalent in each autonomous community), which have little capacity, intention or legitimacy to propose multi-sectoral approaches. This situation should not be regarded as unique to Spain, nor as specific to suicide policies; rather, it is a widespread phenomenon in most European states (10, 62). However, without intersectoral or multisectoral approaches, it is strategically unfeasible to move towards transformative public health proposals with a biopsychosocial focus. A policy framework developed exclusively by public administration sectors with responsibility for healthcare lacks the capacity or expertise to transform fundamental aspects necessary to reduce suicide and promote wellbeing, such as economic and labor dynamics, social protection systems, educational settings, or the work of social organizations that focus on these issues from a more interventionist approach and one that is closer to disadvantaged groups or communities (23, 25–27, 30, 61, 63). Consequently, the social determinants model, if not accompanied by an effective mechanism for intersectoral collaboration, is reduced to a mere conceptual principle. All of this has an impact on the type of measures implemented in regional suicide prevention policies in Spain.

The proposed use of the community-based approach is illustrative. Regional policy echoes the principles of transnational and national policy in the implementation of measures it considers to be community-based. The community-based approach, as discussed, presents conceptual complexity as evidenced in the specialist literature (41, 64). The most socially ambitious proposals recognize that this approach is a model for citizen empowerment, with the aim of enabling citizens to actively take charge of their environments in order to transform social spaces (65, 66). This approach inherently possesses transformative potential. It also involves the active engagement of community actors and spaces in the implementation of actions. The regional suicide prevention policy presents a limited interpretation of this model, equating the ‘community’ aspect with the physical space in which the measure is carried out rather than with the focus of the measure itself.

More generally, when examining the range of preventive and intervention-based measures in regional suicide prevention policy, it becomes apparent that where measures are preventive in nature, the approach is more open to professionals in the social sector, particularly teachers and social services professionals. When it comes to intervention-based aspects, the approach is restricted to health professionals (medicine and psychological sciences), with biopsychological proposals typical of clinical contexts. In this context, a hierarchy within the biopsychosocial model can once more be inferred, in which the cause of suicide is considered to be biopsychological (67), while social factors play a modulating role that may enable prevention but do not provide a cure. This reality has an impact on the self-concept that professionals have regarding their role in suicide, as professionals in the social sector view their role as complementary to or subordinate to that of professionals in the medical field or psychological disciplines (68).

At the same time, and drawing on the notion put forward by Fernández-Vilas et al. (33) regarding the levels of analysis required to understand suicide, the following is concluded: transnational approaches to suicide are situated at the macro and meso levels of analysis of the phenomenon. They ask: what are the structural conditions and immediate determinants of suicide, and how can these be modified to prevent this problem? Regional policies tend to be implemented from the micro level of analysis, and on occasion from the micro to the meso: what individuals are affected by suicide to a larger degree, and what are their characteristics? Thus, suicide prevention policies in Spain’s autonomous communities use the social determinants of health approach as a rhetorical strategy to situate the phenomenon within a biopsychosocial perspective, rather than as a tool for intervention and the design of measures. Through these policies, we cannot expect the material conditions of the population to change, despite the fact that suicide is, to a large extent, constructed as a phenomenon linked to inequalities (22, 40, 42, 61, 69).

This research is not without its limitations. Not all autonomous communities have a specific suicide policy, and in some cases the suicide prevention policy is included within broader mental health policies that have not been analyzed. Future research with a greater focus on both the literature and geographical areas would therefore be warranted. This would enable a comparative analysis across European countries, which would help determine whether the pattern identified in Spanish suicide prevention policy can be generalized to other neighboring contexts.

## Data Availability

The original contributions presented in the study are included in the article/supplementary material, further inquiries can be directed to the corresponding author.
